# Beyond the Intention: Individual-Level Determinants and Intergenerational Differences of Floating Populations’ Actual Settlement Choices in Dongguan, China

**DOI:** 10.3390/ijerph17249194

**Published:** 2020-12-09

**Authors:** Zhiwei Du, Gengzhi Huang, Lixia Jin

**Affiliations:** 1 Key Lab of Guangdong for Utilization of Remote Sensing and Geographical Information System, Guangdong Open Laboratory of Geospatial Information Technology and Application, Guangzhou Institute of Geography, Guangdong Academy of Sciences, Guangzhou 510070, China; chiwai_do@foxmail.com (Z.D.); jlx906@gdas.ac.cn (L.J.); 2 Southern Marine Science and Engineering Guangdong Laboratory (Guangzhou), Guangzhou 510070, China; 3 School of Geography and Planning, Sun Yat-sen University, Guangzhou 510275, China

**Keywords:** settlement choices, individual characteristics, intergenerational differences, floating population, China

## Abstract

The migration process and patterns of floating populations have received continuing attention from scholars and policymakers. In China, however, studies have been focused on the settlement intention of floating populations based on sampling surveys and yielded inconsistent findings. Drawing upon 18,178,167 authentic individual samples of floating populations in Dongguan city, this study contributes to the literature by examining the effect of individual characteristics on the actual resident actions of floating populations, and revealing both the heterogeneity and continuity of their urban residence among four generations (i.e., during the 1960s, 1970s, 1980s, and 1990s). The results show that the proportion of actual resident actions is lower than that reported by previous studies on settlement intentions, and that male, married, middle-aged, more educated, and long-residing members are more likely to choose to stay in Dongguan. Compared to their predecessors, the 1990 cohort reveals significant heterogeneities in their actual settlement choices. The study draws broad implications from the analysis, calling for the equalization of public welfare in Chinese cities and the encouragement of floating populations to sustain long-term residence in the destination cities.

## 1. Introduction

Floating populations, or temporary migrants, are the main components of international migration flows around the world. According to the Organization for Economic Cooperation and Development in 2017, approximately 258 million temporary migrants were living outside their country of origin [1], making significant contributions to their host country’s labor markets and economies. In China, the rural–urban classification is divided with reference to the non-agricultural and agricultural Hukou (household registration) controlled by the central government. Compared to their rural counterparts, urban residents with non-agricultural Hukou are entitled to receive an array of state-provided socio-economic benefits [2]. Floating populations in China refers to those temporary migrants without local Hukou (household registration) at their destinations, with migration from rural to urban locations as their primary trajectory [3,4]. Since the reform and opening-up in the 1980s, and the official relaxation of the Hukou system, millions of people constituting the floating population have poured into urban areas [5]. They have acted as one of the crucial impetuses contributing to the acceleration of rapid urbanization and industrialization in China, especially in the eastern coastal areas [6]. It is estimated that the size of the floating population in China has increased by more than 200 million over the last 40 years, accounting for one-fifth of the total population in 2017 [7].

Significant structural changes have taken place in China’s floating population in recent years. After the growth rate of China’s floating population turned to negative in 2010, the size of the existing floating populations started to shrink in 2015 [8]. According to the National Bureau of Statistics [7], the total floating population in China reached its peak at 2.47 million in 2015 and subsequently declined to 2.44 million in 2017. In addition, it is evident that the willingness of the floating population to reside in cities is not as strong as was previously thought. Instead of settling down in destination cities, the majority of floating populations choose to return to their hometowns or remain in circulation because they are reluctant to surrender the benefits that are tied to their rural Hukou [9,10,11]. Under these circumstances, the actual urban residence of floating populations tends to be more complicated and difficult to predict.

Existing studies have generated and documented a large body of literature on multiple determinants of their settlement intention, diversified trajectories of floating populations’ migration, and differences of the old- and new-generational floating populations. It is commonly recognized that floating population’s settlement intention is tightly tied to their individual characteristics, such as gender, age, marital status, educational attainment, occupation, and residence duration [4,9,12,13,14,15,16]. Regarding generational differences, the new generation are expected to have greater aspirations to remain in destination cities than the old generation had, because the latter retain stronger emotional ties with their places of origin [17,18,19,20,21]. In 2014, China’s central government launched the National New-type Urbanization Plan, which called for a relaxation of the Hukou restrictions in small and medium-sized cities and extended coverage of urban social benefits such as education and health to non-Hukou holders, aiming to achieve a target of relocating an additional 100 million rural migrants to cities and towns by 2020 [22]. Responding to this plan, local governments made significant efforts to remove restrictions created by the Hukou system, in the hope that rural migrants would reside permanently in cities and enjoy the benefits of public services [23,24].

However, studies of floating populations have been constrained by the following three limitations. First, most published research has focused on the settlement intention of floating populations without realizing the fact that there is a gap between their settlement intentions and actual actions [9]. Second, due to the different respondents and surveyed locations, the effect of some individual-level determinants (e.g., gender, age, and Hukou status) on the settlement intention of floating populations remains controversial. Third, some scholars believed the dichotomous approach in comparing generations of floating populations tended to be too simplistic, which resulted in an exaggeration of the intergenerational difference of migrants [19,20]. To fill these gaps, this study aims to examine the following questions: (1) What is the difference between the actual settlement choice of floating populations and their settlement intentions shown by previous studies? (2) How do individual characteristics influence the actual urban settlement of floating populations? (3) How different are the characteristics of actual urban settlement among generations?

Based on 18,178,167 authentic individual samples obtained from the Dongguan Social Security Bureau (DSSB), binary logistic regression models are used to investigate the effect of individual characteristics on actual urban settlement, and the differences of individual characteristic by generations. The study presents an empirical contribution to the literature in that it is the first empirical study using such a large authentic sample to deepen insights into the practical residence of floating populations. Theoretically, it contributes to a better understanding of the distinction between the settlement intentions of floating populations and their actual actions. Policy implications for encouraging the long-term urban residence of floating populations will also be discussed in the conclusion.

## 2. Literature Review

Drawn from international literature, several theoretical perspectives have been proposed to interpret the process of rural–urban and return migration and explain the motivations for urban residence in the context of China. According to the theory of neoclassical economics (NE) and its extensions, migrants choose to settle down or return depending on differentials in incomes and employment opportunities between the origin and the destination [25,26]. Thus, international migration can be seen as the result of geographical differences between supply and demand of labor and income [27,28]. Because of the large urban–rural differences in socio-economic opportunities created by Hukou in China, it is generally recognized that the floating population from rural areas are strongly motivated to stay or reside in cities [5,29,30]. The new economics of labor migration theory highlights temporary migration as a strategy that combines a focus on maximized personal utility and minimized family socio-economic risks [31,32,33]. Migration is regarded as a collective decision of households or families, explaining why migrants often return to origin countries after fulfilling their intentions in destination countries [34]. This perspective is useful to understand the decisions of many floating populations to keep circulating between their places of origin and destinations in China. By remaining in circulation, floating populations can maintain their entitlements such as farming and housing land, compensation for land requisition, and more relaxed birth control in the rural areas while continuing to access economic opportunities and basic public services in cities [10,35,36].

The human capital theory focuses on the role of individual human capital in migration, arguing that highly educated, more skilled, and younger migrants have a greater capacity for finding jobs in cities and adapting to city life [37,38]. Empirical findings show that higher educational attainment and local language proficiency can enhance capabilities to settle among floating populations as well as increase their intentions to integrate into destination cities [19,39]. In contrast, return migrants who leave urban areas and cities tend to be older, less educated, and less skilled; thus, return migrants may have had difficulties finding jobs in cities and adapting to city life. Furthermore, some non-economic factors, such as residential amenities or education for children, are important considerations in household decision-making [35,37,40]. The life-cycle theory postulates that migration is a part of a life-cycle process in which one advances from one stage to another [41,42]. The motivations of floating populations may change especially when they enter specific stages of the life-cycle [19]. There is evidence that many of China’s floating populations decided to remain in or leave urban areas when life events such as marriage or retirement were imminent [33,43].

It is worth noting that most empirical studies on migrant settlement intentions in China have been centered on the settlement intentions of floating populations, taken as an alternative approach to analyze the actual settlement behavior [44], by means of questionnaire surveys and face-to-face interviews. As Zhu and Chen pointed out, however, there is a gap between the floating population’s settlement intentions and their immediate actions [9]. Owing to differences in respondents and surveyed locations, findings reported about levels of settlement intentions of floating populations who intend to make their residence in urban areas vary significantly (see Table 1).

Since not all individuals from, or in, a region reflect the same levels of urban settlement, existing literature has demonstrated that floating populations intending to settle in cities are generally characterized as being unmarried, young-aged, more educated, and staying longer [4,9,12,13,14,15,16,44,45,46]. To be more specific, unmarried and younger floating populations are more likely to settle in the destination cities because they do not need to consider the family members remaining in the source area, or their spouses [9,15]. Compared to their less educated counterparts, those who possess higher levels of education have a significant relationship to migration from rural areas to cities [4,14]. In addition, longer residence duration has a positive effect; if the floating population stay longer, they accumulate more experience and economic stability, and therefore, are more likely to choose to settle down. A possible explanation for this phenomenon is that those with higher educational attainment and more experience are better equipped to earn a living in urban areas [47].

We need to point out that the effect of some determinants (e.g., gender, age, and Hukou status) on the settlement intention of floating populations remains controversial. These discrepancies lack sufficient explanation in the current literature. For instance, some studies demonstrated that the correlation between age and settlement intentions is likely to be an inverse U-shaped curve [14,45], namely, the middle-aged floating population are more likely to stay in cities, while the younger and older ones choose to leave. Conversely, other studies reported that there was a linear relationship as younger people were more eager to settle in the urban areas [21]. Regarding gender, studies found that males can withstand more pressure and tend to have higher incomes; therefore, they were expected to be more likely to stay in cities [12]. In contrast, some researchers supported the observation that females might be the vanguard to settling in cities as they seek economic independence or marry with local urban residents [3,47]. Concerning Hukou status, no significant effects of this characteristic on settlement intentions have been reported because their intentions tend to hinge on equality in welfare benefits rather than the Hukou identity [46].

As a new generation becomes the principal source of China’s floating population [8], it is apparent that attitudes and behavior patterns between the two generations are varied. In contrast to their predecessors, the new generation are more educated and skilled, have higher occupational expectations, stronger desires for social mobility, but are less connected to the rural source areas [17,18,21]. As the dividing line for classifying generations being problematic and somewhat arbitrary, some inconsistencies existed in previous empirical studies [17,19]. Using 1975 as the dividing line, Yue et al. [19] highlighted the sharp differences in the determinants of intentions existing between the new and old generations, involving occupation orientation and socio-economic profiles. Conversely, with a dividing line of 1980, the generational change did not play a significant role in Zhu and Chen’s study [9]. The researchers argued that the contrast between the two generations had been exaggerated [19,20]. Furthermore, Chen and Liu [39] hypothesized that new generation migrants could be more sensitive to social exclusion and the frustration brought about by it; thus, their settlement intention was less marked than that of the old generation migrants.

## 3. Study Area, Data, and Methodology

### 3.1. Study Area and Data Sources

**Figure 1:**
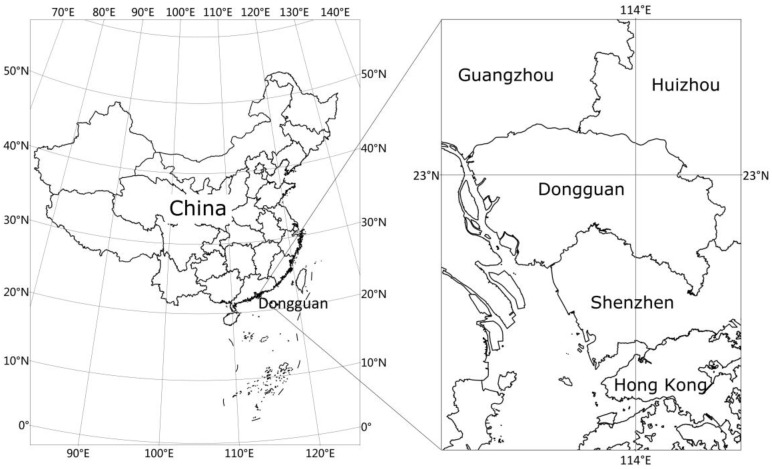
Location of Dongguan.

Dongguan, known as “World’s Factory”, is one of the largest manufacturing bases in the world. It is located in the central section of southern Guangdong province, on the east bank of Pearl River, bordering Guangzhou in the north and Shenzhen in the south (Figure 1). After China’s reform and opening-up, millions of migrants from inland provinces flowed to Dongguan in search of job opportunities. This city has become a representative case of China’s rapid urbanization and is a notable destination city for floating populations. According to the Dongguan Bureau of Statistics [48], there were 6.26 million migrant populations (mainly consisting of floating populations) in 2017, approximately triple the size of the local population. The proportion of migrant populations in the total population was 75.78%, higher than the corresponding proportion in Shenzhen (65.92%) and Shanghai (40.22%). Therefore, research on the actual settlement choices of floating populations in Dongguan has reference value for the African or Southeast Asian urban regions that are still undergoing rapid urbanization.

All data analyzed in this study were generated from the social insurance database, which is established and managed by DSSB. Unlike data derived from questionnaires and interviews, each individual sample in the database is registered with a citizen identity number as unique identification, ensuring that all samples are authentic. The database contains 18,178,167 authentic individuals sampled as parts of floating populations in Dongguan during the years 2008–2015. Each data sample includes information on individual characteristics, including insured state, gender, marital status, age, Hukou status, educational attainment, and residence duration. In China, social insurance provides basic social security for citizens. According to China’s social insurance law, residents are required to participate in social insurance, whether they are employed workers, non-full-time practitioners, or in flexible employment. After excluding registered residents and foreigners working in Dongguan, 18,178,167 samples remained in the database of which 4,178,167 valid samples had the independent variables of marital status and educational attainment available. All individual samples were put into an aggregated format for quantitative analysis.

### 3.2. Variables Selection

For the dependent variable, the insured state displaying “suspend” was coded as 0 and indicates members of floating populations who choose to leave Dongguan, while the insured state displaying “insuring” was coded as 1 and indicates those who stay in the city. The individual characteristics, including gender, marital status, Hukou status, age, educational attainment, and residence duration, were selected as the independent variables. All independent variables and their descriptions are shown in Table 2. In this study, multi-collinearity between independent variables in the model was conducted, and the variance inflation factor (VIF) among the independent variables was all less than 2. Thus, no evidence of multi-collinearity was found.

To investigate the differences between floating populations who choose to stay in or leave Dongguan, all samples are estimated separately in two groups (i.e., the stay group and the leave group) in the descriptive analysis. Binary logistic regression models were used to estimate the effects of characteristics on the actual settlement of floating populations in Dongguan. Owing to incomplete valid samples for the variables of marital status and educational attainment, we first conducted an analysis of four independent variables (gender, Hukou status, age, and residence duration) with all samples in model 1, and subsequently included marital status and educational attainment (with 4,178,167 samples) in model 2.

This study conducted four separate models to investigate the continuity and heterogeneity among different generations in the actual settlement of floating populations (model 3 to model 6, respectively). The generation cohort is defined by age, so the age variable was excluded from these four models. Since the dichotomous approach of dividing generations is inadequate and simplistic [17,19], three dividing lines (1970, 1980, and 1990) are employed to differentiate four generations; namely, the 1960 cohort (born 1960–1969), the 1970 cohort (born 1970–1979), the 1980 cohort (born 1980–1989), and the 1990 cohort (born 1990–1999). These four cohorts make up the vast majority of floating populations in Dongguan, accounting for 96.98% of all samples in our investigation.

Additionally, the odds ratio (OR) is used to estimate the correlated relationship between the individual characteristics and actual urban residence. If the OR > 1, they are positively correlated, indicating that the presence of individual characteristics will raise the likelihood of actual urban residence. Conversely, if the OR < 1, then they are negatively correlated, meaning that the likelihood of actual urban residence will be reduced.

## 4. Empirical Findings

### 4.1. Descriptive Analysis

Table 3 shows descriptive statistics for the independent variables. As expected, the results indicate the unsettled nature of China’s floating population, with 4,084,511 samples in the stay group compared to 14,093,656 samples in the leave group. In contrast to results of settlement intention in previous literature, this study shows a more negative result, suggesting that the overall stay rate (the proportion of stay group in all samples) of the floating population in Dongguan is only 22.47%. Moreover, significant intergenerational differences exist: the stay rates of the floating population decreased with generational change (from 33.03% of the 1960 cohort, to 26.45% of the 1970 cohort, and to 15.69% of the 1980 cohort), but the 1990 cohort shows a slightly higher proportion with its stay rate being 23.86%.

In all samples of floating populations, the proportion of males (56.47%) was larger than that of females (43.52%). It is difficult to judge the effect of gender on actual settlement choice because the proportions in the two groups are analogous: 43.23% of the floating population who chose to reside in Dongguan and 13.25% who chose to leave are males, compared to 34.31% and 9.22%, respectively, being females. Similarly, the proportions of the non-agricultural floating population in the two groups (4.54% in the stay group and 11.69% in the leave group) are both smaller than those of the agricultural floating population (17.93% in the stay group and 65.84% in the leave group). Thus, the effect of gender and Hukou status on floating populations’ actual settlement choice needs to be further examined using a binary logistic regression analysis.

For marital status, the proportion of married and unmarried are roughly equal in the leave group (27.04% vs. 23.51%), while the proportion of them in the stay group presents a remarkable difference (36.64% vs. 12.82%), indicating that engagement or marriage is a crucial characteristic of the floating population who choose to reside in Dongguan. As for age, the mean ages of the stay and the leave group were 33.59 years and 30.65 years, respectively, with the former being an average of 2.94 years older. This finding reflects that the members who stayed are mainly from the middle-aged group.

Compared to those who leave Dongguan, the staying floating population is more likely to be more educated and have longer residence duration. Regarding educational attainment, 54.38% of floating populations achieved the education level of junior secondary school, followed by 28.78% who completed senior secondary school. These results indicate that Dongguan’s floating population structure is dominated by a moderately educated industrial population. In terms of residence duration, 53.76% of all samples fell into the category of “less than 1 year,” which reflects the unstable nature of the floating population. Additionally, the increase in residence duration corresponds to the disproportionate decline in the proportion of floating population in the leave group, with 49.03% in “less than 1 year”, 14.75% in “1–2 years”, 6.98% in “2–3 years”, 3.58% in “3–4 years”, 1.83% in “4–5 years”, and 0.92% in “5–6 years”. It is noteworthy that 4.48% of all samples demonstrated residence in Dongguan for more than six years, with the proportions of this category being 4.04% and 0.44% for the stay group and the leave group, respectively.

### 4.2. Individual-Level Determinants of Actual Settlement Choices in Dongguan

The results of the logistic regression reported in Table 4 show that models 1 and 2 are statistically significant. Compared to all samples in model 1, only the age variable is not significant in model 2; whereas, other independent variables are significantly associated with actual urban residence. As expected, the floating population who chose to stay in Dongguan is closely related to their individual characteristics, namely, being male, married, middle-aged, more educated, and long-residing.

In models 1 and 2, setting the male category as reference, the ORs associated with female were 0.930 and 0.916, respectively, indicating that the female floating populations are less likely to choose residence in Dongguan than the males. This result not only supports previous studies [12,47] that males are more likely to consider settlement in the cities, but also reveals the phenomenon that the actual urban residence of the floating population is strongly tied to its industrial structure. In the 1990s, Dongguan’s floating population was dominated by females [49] because they were regarded as being “more docile, scrupulous and nimble-fingered,” and therefore more suitable in labor-intensive industries, such as textiles, electronics, and shoemaking. Accompanying the economic transition toward capital- and technology-intensive industries after 2008 [50], males became the majority in Dongguan’s demographic structure by 2010, with 56.14% in the total population in 2017 [48].

For Hukou status, the ORs associated with agricultural members is shown were 0.284 in model 1 and 0.479 in model 2, when the non-agricultural category is set as reference. These statistics indicating that floating populations with non-agricultural Hukou are more likely to seek residence in Dongguan. Our study confirms the importance of Hukou in floating populations’ actual settlement choices. Moreover, compared to those who are married, the unmarried floating populations’ OR was 0.709 times as likely to reside in Dongguan. The results also suggest that getting or being married is an important factor for the floating population choosing to reside in Dongguan, consistent with the perspective of the life-cycle theory. With reference to the NE theory, it is clear that although China’s rural–urban differences in income and employment opportunities are narrow, floating populations have requirements for equal access to urban public services that still differ according to Hukou status, especially in social housing, children’s education, pensions, and medical care.

**Figure 2:**
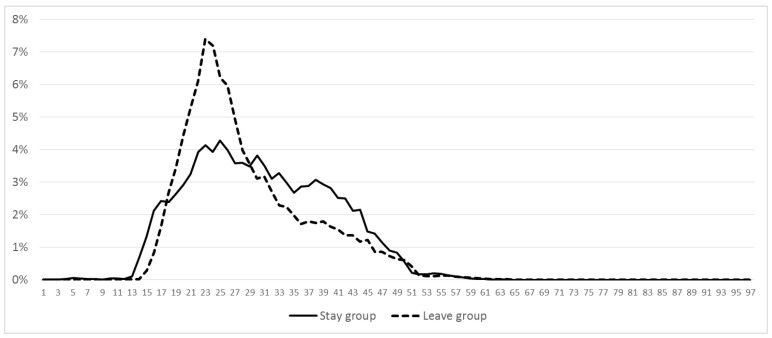
Age and proportion of the floating population.

When it comes to age, the results in model 1 show the OR > 1, indicating that increasing age significantly increases the likelihood of staying in Dongguan. In addition, both coefficients for “age” and “age square” are statistically significant but the quadratic term is significantly negative (Coef. = −0.092), suggesting that the relationship of age and actual urban residence is inverse U-shaped; that is, those middle-aged floating populations are more likely to settle down. The result also interprets the aforementioned intergenerational difference: the 1960 cohort and the 1990 cohort have higher proportions (33.03% and 23.86%, respectively), compared to 26.45% and 15.69% in the 1970 cohort and the 1980 cohort, respectively. Another noticeable finding is that the age range of 24 to 36 years has the largest proportion of floating population who choose to stay in Dongguan, accounting for 47.84% of all samples in our investigation (Figure 2). In contrast, the largest proportion of the floating population who leave Dongguan is in the range 22 to 28 years old. Our study has reached inconsistent with findings with respect to the young-aged positively contributes to settlement intention [1,3], probably because the younger members constitute the 1990 cohort that shows significant heterogeneities in actual settlement choices in comparison to their predecessors.

Consistent with previous studies of urban settlement intention [9,15], educational attainment and residence duration are positively correlated with actual residence in cities. Using the “primary school or below” category as reference in model 2, the OR of the “junior secondary school” is 0.977 times that of the reference group, while the ORs of the “senior secondary school,” the “junior college/university,” and the “postgraduate or above” are 1.186 times, 1.830 times, and 3.098 times, respectively. Since the ORs steadily increases with the improvement of education levels, these results underscore educational attainment as a determinant of actual urban residence. Moreover, the effect of residence duration on the actual residence of floating populations in the cities is significantly positive, which supports our initial hypothesis. Compared to the category of “less than 1 year,” those who choose to stay in Dongguan for more than six years are 157.485 times (model 1) and 297.213 times (model 2) more likely to have actual urban residence. Related to the human capital theory, long residence in Dongguan can improve human-capital accumulations (for example, skills for operating machines and Cantonese proficiency), which strong contributes to floating populations’ actual settlement choices.

### 4.3. Intergenerational Differences in Actual Settlement Choices in Dongguan

The regression results of the four models differentiated by generations are shown in Table 5. All selected independent variables are significant at the 1% level, and their effects on actual urban residence are consistent with all samples in model 1.

On the one hand, our survey results do confirm that there are significant heterogeneities among different generation migrants. Significant intergenerational differences exist in gender: setting the male category as reference, the ORs associated with females of the 1970 and the 1990 cohorts are higher than 1 (1.074 and 1.004, respectively), compared to 0.902 and 0.837 for the 1960 and the 1980 cohorts, respectively. The results indicate that the likelihood of females born in the 1970s and 1990s staying in Dongguan was higher than that of females born in the 1960s and 1980s. Corresponding to age differences, the increase in age significantly enhances the likelihood of staying in cities for the three generations (the 1960s, 1970s, and 1980s), but the corresponding likelihood of the 1990 cohort tends to decrease, reflecting prominent dichotomous differences in the actual urban residence of floating populations.

On the other hand, several lingering continuities exist among the different generations. Reflecting the findings in model 1, additional years of residence duration increases the likelihood of residence in Dongguan, which supports the effect of long-term duration in promoting urban settlement. More interestingly, the likelihood of actual urban residence presented a descending trend by generation. For example, the OR associated with four to five years declined from 41.118 for the 1960 cohort, to 37.143 for the 1970 cohort, to 35.177 for the 1980 cohort, and then to 24.424 for the 1990 cohort. Additionally, the Hukou status of the floating population in four generations revealed significantly lingering similarities, showing that the likelihood of actual urban residence increased according to a change in generation. Setting the non-agricultural Hukou category as reference, the ORs associated with agricultural Hukou for the 1960, 1970, 1980, and 1990 cohorts are 0.242, 0.298, 0.307, and 0.853, respectively, indicating that the effect of Hukou status on hindering residence in cities is diminishing. Deserve to be mentioned that the 1990 cohort is shown to have less concern about entitlements attached to the agricultural Hukou, manifesting significant heterogeneities compared with their predecessors.

## 5. Discussion

The results of this study provide evidence to confirm the existence of a gap between the floating populations’ intention to settle in the cities and their immediate actions, as proposed by Zhu and Chen [9]. As argued by some scholars [4,11], the intention of floating populations to stay in cities appears not to be as strong as previously thought. Instead of abolishing the Hukou system, it is preferable to advance new-type urbanization by promoting social integration of floating populations and strengthening their sense of belonging in urban areas, especially for the 1990 cohort who demonstrate relatively high levels of identification with urban society. This study contributes to solving the dispute concerning the role of some individual-level determinants (e.g., gender, age, and Hukou status) on settlement intentions by such a large population sample. It not only transcends the dispute about whether the relationship between age and actual urban settlement is linear [21] or inverse U-shaped [14,45], but also reveals the group aged 24 to 36 who exhibited a greater propensity to settle down in urban areas.

Previous studies have demonstrated that the settlement intention is shaped by a series of socioeconomic factors [4,16,46,51,52,53,54], including household factors (e.g., family members, housing type, and household income per capita), occupational status (e.g., employed sectors, job duration, and monthly wage), and social factors (e.g., physical health, relationship with local residents, and migration experience). However, individual samples taken in this database did not contain these variables and therefore could not be taken into account for the effects of socioeconomic factors on floating populations’ actual urban settlement. Another limitation of this study is the lack of consideration of the interactive effects of individual determinants and geographical contexts (place of origin and choices of destination) [16,55] on actual settlement choices. The next step in this study is to jointly model such factors to understand how individual determinants and geographical contexts interactively shape actual settlement choices of floating populations. Finally, it should be noted that our findings cannot be simply generalized at the national level as the samples were obtained only in Dongguan. Although Dongguan is an ideal city for the study of floating populations’ settlement in cities in the eastern region of China, it might restrict the generalization of our findings to extend research to cities in the central and western regions given remarkable regional differences existing in the country. There is a need to expand this study to other urban areas to examine whether the results can be generalized at the national level.

## 6. Policy Implications

By revealing the complicated individual characteristics and intergenerational differences of the actual residence of the floating population, our empirical analysis sheds light on the current “people-oriented urbanization” in the policy design of local governments. In the Key Tasks for New Urbanization Construction in 2019 [56], the central government has taken measures to ease restrictions for rural residents applying for urban Hukou, to allow more people to enjoy public services, and to increase infrastructure spending in cities. The above studies related to the actual settlement choices of floating populations raise three implications for understanding their future trends and for relevant policy-making.

Local governments need to emphasize the role of the floating population’s long-term residences in promoting sustainable urbanization. Improved equity in society and quality of living environments are beneficial for facilitating the ability and willingness of the floating population to sustain long-term residence in the destination cities. In an immigrant city like Dongguan, policymakers should grant welfare entitlements and ensure health equity for industrial migrants who made substantial contributions to the industrialization and urbanization of this city.

All floating populations (including non-Hukou holders in China) should be integrated within an equal public welfare system. Consideration should be given to residence duration, and the welfare systems should be built by local states. In this type of system, the floating population should be gradually permitted to enjoy the benefits of public welfare, especially basic health care, social housing, and children’s education, attached to their years of residence.

More attention should be paid to the main group of inhabitants who are associated with the economic and demographic change. In the case of Dongguan, these members are characterized as male, married, aged 24 to 36, more educated, and belonging to the new generation born after the 1990s. With advances in big data analytics, relevant departments and organizations can utilize a huge magnitude of data concerning access to provide public services and guide population health management in relation to floating populations.

## 7. Conclusions

Using 18,178,167 authentic individual records from Dongguan, the study investigated the effect of individual characteristics on the actual urban residence of the floating population and demonstrated their heterogeneity and continuity by generations in actual urban residence. The main findings are summarized as follows:

The proportion of actual resident actions was found to be lower than the settlement intentions found in previous studies [4,19,44,45,46], with only 22.47% of the floating population choosing to stay in Dongguan as the destination city. Considering the intergenerational differences, the floating population of the 1990 cohort have intergenerational discontinuity in actual settlement choice compared to their predecessors (the 1960, the 1970, and the 1980 cohorts), which showed that the stay rates decreased with generational change.

Inspired by controversies in existing literature, the logistic regression analysis in the present study reveals that the actual urban residence of floating populations is closely related to their individual characteristics, with those who are male, married, middle-aged, more educated, and long-residing having a slightly higher proportion choosing to stay in the destination city. Our findings demonstrate that the relationship of age and the actual urban settlement is inverse U-shaped with those middle-aged floating populations are more likely to long-term settle down.

Furthermore, the study found that there are significant differences and similarities among four generations. While gender and age presented significant heterogeneities, residence duration and Hukou status showed some lingering continuities. This study also demonstrated that there are lingering continuities of individual characteristics on actual urban residence among the floating population who were born in the 1960s, 1970s, and 1980s, but that the 1990 cohort has indeed significant heterogeneities when compared to its predecessors. Thus, we suggest that the 1990 might be a suitable dividing line to divide the new and old generations.

## Figures and Tables

**Table 1:** Some survey results of the settlement intentions of the floating population.

Authors	Survey Areas	Survey Year	Valid Responses	Survey Method	Level of Settlement Intentions
[4]	Five cities in Fujian Province	2002	243	Nationwide survey	20.6%
[19]	Shenzhen	2005	1598	Questionnaire interview	41.5%
[45]	Shenzhen	2008	626	Questionnaire interview	45.7%
[44]	Twelve cities in China	2009	2398	Face-to-face interviews	54.56%
[46]	China’s 31 provinces	2012	156,705	Questionnaire interview	60.2%

**Table 2:** Descriptions of the independent variables.

Independent Variables	Description
Gender	Male = 0; female = 1
Marital status	Married = 0; unmarried (single/divorced/widowed) = 1;
Hukou status	Non-agricultural = 0; agricultural = 1
Age	Mean (years)
	The 1960 cohort; the 1970 cohort; the 1980 cohort; the 1990 cohort
Educational attainment	Primary school or below; junior secondary school; senior secondary school (vocational secondary school); junior college/university; postgraduate or above
Residence duration	Less than 1 years; 1–2 years; 2–3 years; 3–4 years; 4–5 years; 5–6 years; more than 6 years

**Table 3:** Descriptive statistics of independent variables in two groups.

Independent Variables		Stay Group	Leave Group	All Samples
Gender	Male (%)	13.24	43.23	56.47
	Female (%)	9.22	34.31	43.53
Marital status *	Married (%)	36.64	27.03	63.67
	Unmarried (%)	12.82	23.51	36.33
Hukou status	Non-agricultural (%)	4.54	11.69	16.23
	Agricultural (%)	17.93	65.84	83.77
Age	Mean age (years old)	33.59	30.65	31.31
	The 1960 cohort (%)	4.66	9.45	14.11
	The 1970 cohort (%)	6.83	18.98	25.82
	The 1980 cohort (%)	6.83	36.70	43.52
	The 1990 cohort (%)	3.23	10.31	13.54
Educational attainment *	Primary school or below (%)	2.79	2.75	5.55
	Junior secondary school (%)	24.16	30.22	54.38
	Senior secondary school (%)	14.48	14.30	28.78
	Junior college/university (%)	6.76	4.34	11.10
	Postgraduate or above (%)	0.13	0.07	0.19
Residence duration	Less than 1 year (%)	4.73	49.03	53.76
	1–2 years (%)	3.72	14.75	18.48
	2–3 years (%)	3.08	6.98	10.06
	3–4 years (%)	2.64	3.58	6.22
	4–5 years (%)	2.28	1.83	4.11
	5–6 years (%)	1.98	0.92	2.89
	More than 6 years (%)	4.04	0.44	4.48

Notes: * Only 4,929,175 valid samples for this independent variable.

**Table 4:** Regression results on individual characteristics and actual urban residence.

Independent Variables	Model 1 (N = 18,178,167)		Model 2 (N = 4,929,175)	
	Coef.	OR	Coef.	OR
Gender (ref = male)				
Female	−0.073 ***	0.930	−0.088 ***	0.916
Marital Status (ref = Married)				
Unmarried	/	/	−0.344 ***	0.709
Age	0.123 ***	1.131	0.006 ***	1.006
Age square	−0.092 ***	0.912	−0.001	0.999
Hukou status (ref = non-agricultural)				
Agricultural	−1.260 ***	0.284	−0.736 ***	0.479
Educational attainment (ref = primary school or below)				
Junior secondary school	/	/	−0.023 ***	0.977
Senior secondary school	/	/	0.171 ***	1.186
Junior college/university	/	/	0.604 ***	1.830
Postgraduate or above	/	/	1.131 ***	3.098
Residence duration (ref = less than 1 year)				
1–2 years	1.124 ***	3.076	2.505 ***	12.239
2–3 years	1.786 ***	5.963	3.337 ***	28.141
3–4 years	2.418 ***	11.226	3.730 ***	41.677
4–5 years	3.039 ***	20.875	3.983 ***	53.687
5–6 years	3.595 ***	36.401	4.294 ***	73.240
More than 6 years	5.059 ***	157.485	5.694 ***	297.213
Constant	0.101 ***	1.105	−3.010 ***	0.049
Log likelihood	−7,296,257.5	−7,296,257.5	−2,593,412.3	−2,593,412.3

Notes: *** *p* < 0.01.

**Table 5:** Regression results on individual characteristics and actual urban residence by generations.

Independent Variables	Model 3: The 1960 Cohort (N = 2,564,079)		Model 4: The 1970 Cohort (N = 4,692,926)		Model 5: The 1980 Cohort (N = 7,911,905)		Model 6: The 1990 Cohort (N = 2,461,864)	
	Coef.	OR	Coef.	OR	Coef.	OR	Coef.	OR
Gender (ref = male)								
Female	−0.102 ***	0.902	0.072 ***	1.074	−0.177 ***	0.837	0.004	1.004
Hukou status (ref = non-agricultural)								
Agricultural	−1.421 ***	0.242	−1.209 ***	0.298	−1.181 ***	0.307	−0.159 ***	0.853
Age	0.141 ***	1.151	0.181 ***	1.199	0.197 ***	1.218	−0.020 ***	0.979
Residence duration (ref = less than 1 year)								
1–2 years	1.474 ***	4.370	1.387 ***	4.005	1.397 ***	4.045	1.023 ***	2.782
2–3 years	2.278 ***	9.764	2.168 ***	8.747	2.174 ***	8.801	1.267 ***	5.407
3–4 years	2.992 ***	19.918	2.903 ***	18.229	2.911 ***	18.381	2.422 ***	11.278
4–5 years	3.716 ***	41.118	3.615 ***	37.143	2.560 ***	35.177	3.195 ***	24.424
5–6 years	4.367 ***	78.875	4.263 ***	71.076	4.128 ***	62.057	/	/
More than 6 years	6.091 ***	441.713	5.755 ***	315.777	5.029 ***	152.912	/	/
Constant	−8.295 ***	0.002	−8.457 ***	0.002	−7.216 ***	0.001	−0.951	0.386
Log likelihood	−1,000,473.9		−1,783,067		−2,603,616.7		−1,280,124.3	

Notes: *** *p* < 0.01.
